# Cerebro-Spinal Meningitis

**Published:** 1864-07

**Authors:** E. Y. Yager

**Affiliations:** Chillicothe, Livingston Co., Mo.


### ARTICLE XXVI.

### CEREBRO-SPINAL MENINGITIS.

Letter Read before the Chicago Medical Society, By JOHN BARTLETT, M.D., from DR. E. Y. YAGER, of Chillicothe, Livingston Co., Mo.

MAY 8, 1864.

DR. BARTLETT,—In compliance with your request, I will try and give you a rough sketch of cerebro-spinal meningitis, as it occurred in this county. Were I to consult my own feelings,

I should wait until more facts could be gathered, and give a more extended report.

Cerebro-spinal meningitis first made its appearance in this county the 1st of Feb., 1862, and continued until the 1st of May following. Except one case in Nov. and two in Dec. 1863, no more cases were met with until Jan. 1864.

The first cases occurred in Feb. 1862, in the 23d Reg. Mo. Vol., stationed in Chillicothe. Five soldiers were attacked in the morning, after having stood picket guard all night in the edge of a prairie. The snow was several inches deep. A storm had commenced the day before, which continued through the night. The air was damp and chilly, the wind from the East. A dense fog arose the evening before and continued until late next day. Of the five attacked three died in less than 48 hours,—two recovered after a protracted convalescence. I did not see these cases and cannot give their history. I heard they all became delirious in a very short time, and *the four* died comatose. The treatment I did not ascertain, except that no blood was drawn. Several others of the same regiment were attacked, and the majority died in a few hours.

Other cases occurred in the 3d Reg. M. S. M. Cavalry and a Battalion of Militia Infantry, stationed in this town. I saw three cases belonging to the 3d Regiment treated by Dr. MAY, and one of the same regiment by Dr. COOPER. The three cases treated by my friend Dr. M., were attacked the 1st of April, with chills and severe headache; high fever followed with increased headache. They complained in a short time after the attack, of soreness and pain in the muscles of the neck and jaws. In six hours the patients were delirious, with heads drawn back and rotated from side to side; tenderness along the spine; head hot; tongue coated with thick, dark brown fur in middle, and redness along tip and edges. The bowels were constipated. Two recovered, and one died in less than 60 hours. The treatment was commenced with a purge of calomel combined with ipecac, and followed by small and frequent doses of the same medicines. A blister was applied to the nape of the neck, extending down the spine some distance. The two that survived, had a protracted convalescence. They fell into a low typhoid after a few days and did not leave the bed for weeks. One never recovered perfectly—still being feeble. Dr. COOPER'S case was very similar to the above cases, except a few hours after the attack, he had several spasms. He died comatose, 60 hours after the attack.

During February, March, and April, quite a number of citizens were affected, and several died. By far the heaviest mortality was among persons confined in the guard house for political offenses. My information is that not one recovered. I think there were five deaths in the guard house in a month. There never being more than a dozen or fifteen in the prison at one time, makes this a very heavy mortality.

But few cases occurred in this county outside of the town and township. The counties North suffered some during March and April. I have no data by which I can form an estimate of the number of deaths in Grundy, Mercer, and Harrison counties where it prevailed, but I learned from citizens that it was not very fatal.

Dr. J. E. CADLE, living in the North-western portion of this county [Livingston], treated five cases in March and April. Of the five three were girls, and two boys. Two were four years old, two six, and one twenty. Two girls recovered after a long convalescence,—one lost an eye, the other the speech. The three fatal cases survived from 4 to 12 days. These patients were taken with chill and headache and followed by fever, delirium, stiffness in the muscles of the neck and jaws; dilitation of the pupils in three, and contraction in two; tenderness from nape of neck to seventh cervical vertebræ; bowels constipated. The delirium was very violent after a few hours. Treatment was antiphlogistic,—blood-letting freely, calomel, tart. em't. and spirits nitre, counter-irritation to nape of neck and along spine.

He had also two cases in Dec. 1863, one a boy seventeen, and the other a girl, eight. Treated girl as the five. The boy was moribund when seen, and no medicine was given. He died in less than 24 hours,—the girl in 48.

In Jan. and Feb. 1864, Dr. C. treated thirteen cases of cerebro-spinal meningitis; seven were boys and six girls,—all between four and sixteen years old. Eleven were fatal and two survived.

The attack was ushered in by a chill and violent pain in the head, back, and extremities,—followed by fever in most cases and delirium; tenderness along spine and nape of the neck; difficulty in swallowing; head very hot, drawn back very much and rotated from side to side. In three cases there was no reaction, the patients died comatose in a few hours. Bowels in all costive; pulse weak and irregular; pupils dilated. Nine died in 24 hours; one the fifth week, and one the sixth. The two that recovered had a very protracted convalescence. The two that survived until the fifth and sixth week, after five days sunk into a low typhoid condition, and died comatose. The nine that died within 20 hours, were treated as the five cases in 1862, with the addition of bathing feet in warm water and mustard to extremities. The other five were not bled but treated otherwise the same.

The first case I saw was a boy 18 years old, the patient of another gentleman, who was absent from town when I saw it. The history I had of the case from the family was, that four days before he had a chill with severe headache and followed by high fever and violent delirium. The head was very hot and drawn back considerable. Tenderness along the spine; difficult swallowing; bowels constipated. The patient continued in this condition until the third day, when coma gradually came on. When I saw him four hours before death he was in a profoundly comatose condition, with stertorous breathing; pupils dilated; head drawn back very much; pulse one hundred forty-five, and very weak.

The second case was a brother of the above patient, aged 9 years. I was called Feb. 10, 1862, in the afternoon. The first patient died the night before. I found the boy suffering the most agonizing pain in the muscles of the right thigh, which changed to the patella in a few minutes, and then to the foot. It continued to change its location, never remaining longer than an hour in any locality. Face was ghastly pale and *pinched;* complained of some pain in back of head which was hot and drawn back; tenderness from the occiput to seventh cervical vertebræ; pulse weak and irregular; some difficulty in moving head in consequence of the stiffness of neck. Bowels costive; tongue slightly coated in centre and red around tip and edges; pupils slightly dilated. The first symptom was pain in the arm, which had changed to muscles of the thigh. Patient never complained of chilly sensations at any time. Opened bowels twice with calomel and jalap, and then gave small doses of calomel and ipecac, with two grs. of pulvis Doveri, every two and a-half hours; applied mustard to back of the neck and down the spine. Saw him Feb. 11th, 9 A.M. Bowels moved twice early in the night. Rested little during the night; more headache; less general pain. Other symptoms as the night before. Applied blister to nape of the neck and along spine. Continued treatment. 5 P.M., patient little changed—mouth drawn to the left. Pulse rather stronger than before; face not quite so pale; continues to complain of some pain in the head. Blister was not used until in the afternoon.—Continued treatment. 12th, 9 A.M., patient slept two or three hours during the night; feels better; head and mouth less drawn; has but little use of the right leg; pulse has more volume. Continued powders every two hours. 8 P.M., patient doing well; took little broth; head and mouth less drawn; pupils less dilated. Continued every four hours the powders. Gave pulvis Doveri 2½ grs. at 9 o'clock. 9 A.M. next morning, saw patient; doing well; has a little use of the right leg. Gave powder of ipecac, Dover and nit. pot. Saw patient morning 14th; doing well; ordered powders given every five hours. Feb. 15th, patient convalescing; has little use of leg. This patient took a little quinine for several days after. He left his bed three weeks afterward. He used a cane for three or four weeks after leaving the bed.

The third case was the mother of the boys and aged 45. Her case was very much like the second. She was taken with chilly sensations, slight headache, and pain in the arm. The left arm became weak as the leg of the boy. She was attacked Feb. 11th. The treatment was the same, with the same result. She was confined two and a-half weeks to bed. The arm gradually recovered its strength.

The fourth case was a servant woman, about 50 years of age. The woman was taken with chilly sensations and headache during the afternoon of Feb. 16th. The headache increased and fever set in towards evening. She spent a very uncomfortable night, suffering with pain in the head, feet, and hands, with soreness in the muscles, generally and especially in the muscles of the neck and back. Through the day following she continued to grow worse. I was called at 11 P.M., on the 17th to see her. I found her with the head drawn slightly back; pupils dilated; pulse frequent and weak; tongue coated, soft, and tremulous; bowels constipated; skin soft and relaxed. The most prominent symptom was the excessive sensitiveness over the entire body. There was not a fibre that was not most exquisitively painful on the slightest touch. The pulse was barely felt in consequence of the extreme sensibility. She suffered great pain in swallowing, and had difficulty in opening the mouth; shutting the eyes gave pain. The speech was very thick and imperfect. I could scarcely convey an idea of the extraordinary sensitiveness over the entire body. Gave ℥i. of oil to be repeated every two hours until the bowels moved well, and ordered an injection of sul. mag. after second dose of oil if the bowels did not move in one hour. The bowels moved twice four hours after first dose of oil. Give 1-3d gr. morphia, to be repeated every two hours unless she became comfortable, when it was to be discontinued. 17th, 8 A.M., scarcely so much sensitiveness,—otherwise the same. Used morphia every two hours up to present. Give calomel 4 grs., pulvis Doveri 12, ipecac 3, to be continued every two hours. 18th, 11 A.M., patient unchanged. Order treatment continued. 19th, 10 A.M., slept a little during the night and feels some better; pulse can be felt without giving much pain. Continued prescription with 6 grs. less of Dover powder and 2 of calomel. Her bowels moved twice during the night. The evacuations were of a dark offensive nature. The patient during the afternoon gradually became stupid, until profound coma occurred, in which condition she died in the forenoon of the 20th.

I have not seen but two cases of cerebro-spinal meningitis this year. It has been almost entirely confined this year to the N. W. portion of the county. The two cases were almost identical. They were boys aged 8 and 10. Both were attacked Feb. 8th, in the afternoon, with a chill and pain in the head and stiffness of the muscles of the neck and jaws. After the chill wore off fever came on which did not run very high. During the night they began to complain of pain through the muscles generally, especially in the hands and feet. The morning found them with more pain in the head and very restless. Pain continued to increase during the forenoon, and by 3 o'clock the restlessness was so great that it was difficult to restrain them from leaving the bed. The older boy was seen by Dr. J. S. WILLIAMS on the afternoon of the 9th. He gave him a purge, calomel combined with a small portion of ipecac, to be followed after purgation with calomel in small doses combined with ipecac and pot. nit., applying ice freely to the head. The younger boy did not have any physician on the 9th. His father gave him three doses of calomel during the night combined with ipecac, applying a blister to the back of the head and neck. I saw both boys next day. The older boy was lying calm and composed; face extremely pale; pulse very quick and weak, intermitting once or twice in ten beats; head very hot, notwithstanding a sack of crushed ice had been to it for hours; head drawn back to an angle of 20 degrees; pupils dilated, and most exquisite pain given when any pressure was made along the upper half of the spine. Complains of severe pain in the head and in the right pectoral muscle. Bowels had been moved by the purgative given by Dr. W., during the night. The operations were said to be dark and offensive. The family report that he has been furiously wild from the early part of the night until 10 o'clock this morning, when he began to calm down. Ordered dry cupping along the back of the neck and down the spine, to be followed in six hours by blister if it did not relieve the tenderness. Gave small doses of calomel combined with ipecac and pot. nit. every two and half hours. Feb. 11th, patient became restless and delirious soon after my departure the day before. The cups relieved him some. He continued delirious and restless until the blister was used. After it drew he seemed some composed and slept two hours. Head still drawn back; pulse more regular; more rational than during the night; less heat and pain about head. Continued treatment. Feb. 12th, patient rational this morning; had restless and delirious periods during the night; head less drawn back; pupils continue dilated. Continued treatment. 13th, 11 A.M., patient continues to improve. Ordered powders given every four hours. 14th, patient doing well. Continued powder every six hours. This patient had a little quinine for three or four days following. He continued to improve slowly and was able to leave the house in three weeks.

The course, result, and treatment was so much like the above that it is not necessary to speak of the younger boy's case.

This county (as is North Missouri generally), is noted for health. We have some intermittent and remittent fever in autumn and pneumonia in the winter. But the mortality, except in cerebro-spinal meningitis, is small. The epidemic of both 1862 and '64 was preceded by very cold weather. You will observe that no case occurred until after the commencement of the thaw. The wind was generally from the East and very chilly. There was a dense fog preceding both epidemics. At the time of its prevalence in 1862, there were quartered near twelve hundred soldiers in different parts of the town. They were very much crowded; whole companies in houses that were not capable of accommodating more than fifteen. Measles prevailed to an alarming extent among soldiers and citizens. There was a very large mortality attending the epidemic of measles, and later in the season pneumonia typhoides was very prevalent. I think there was no erysipelas that season. The quarters and hospitals were in the worst possible condition. Following the epidemic of 1864, we have erysipelas and pneumonia typhoides. Scarlatina of a mild form preceded the last epidemic. By far the larger number of cases occurred in the N.W. part of the county. The majority of the cases reported by Dr. Cadle were children attending the same school. It is said more than half of the pupils died in less than a month. The schoolroom was habitually kept very warm. It was a country school, and of course the house was very deficiently ventilated. It has been observed by all physicians that the diseases generally have assumed an asthenic character since the first epidemic. I have no knowledge of the occurrence of a single case of cerebro-spinal meningitis in dry weather. It was invariably muddy and damp, the wind usually from the East. During the great prevalance both in Feb. 1862 and '64, there was a very peculiar feeling impressed on every one by the atmosphere. It was remarked the first epidemic. Persons predicted its occurrence in Feb., 1864, before it appeared, by the peculiar condition of the atmosphere. Almost every one felt rather full about the head. Epistaxis was common during both epidemics.

---

ARTICLE XXVII.

